# Supplementary material for: Homopolar Chemical Bonds Induce In‐Plane Anisotropy in Layered Semiconductors
Source: Small Sci. 2024 Jun 3;4(9):2400226. doi: 10.1002/smsc.202400226 (PMC11935091; doi:10.1002/smsc.202400226)
Supplement: Supplementary file 1 — Supplementary Material [file SMSC-4-2400226-s001.pdf]

Supporting Information

Homopolar Chemical Bonds Induce In-Plane Anisotropy in Layered Semiconductors

Jieling Tan[1], Jiang-Jing Wang[1], Hang-Ming Zhang[1], Han-Yi Zhang[1], Heming Li[1,2], Yu Wang[2], Yuxing Zhou[3], Volker L. Deringer[3]*, Wei Zhang[1]**

[1]Center for Alloy Innovation and Design (CAID), State Key Laboratory for Mechanical Behavior of Materials, Xi'an Jiaotong University, Xi'an, 710049, China

[2]School of Physics, Xi'an Jiaotong University, Xi'an, 710049, China

[3]Inorganic Chemistry Laboratory, Department of Chemistry, University of Oxford, Oxford, OX1 3QR, UK

*Emails: j.wang@mail.xjtu.edu.cn, volker.deringer@chem.ox.ac.uk, wzhang0@mail.xjtu.edu.cn

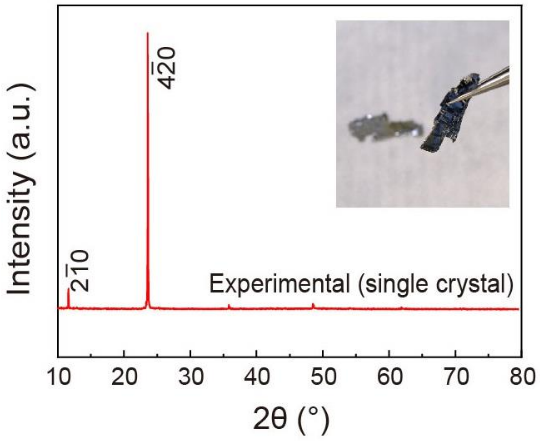

Figure S1. Experimental XRD pattern of bulk m-GaTe. The inset shows an optical image of the bulk sample. There are two strong peaks corresponding to the (2$\bar{1}$0) and (4$\bar{2}$0) planes.

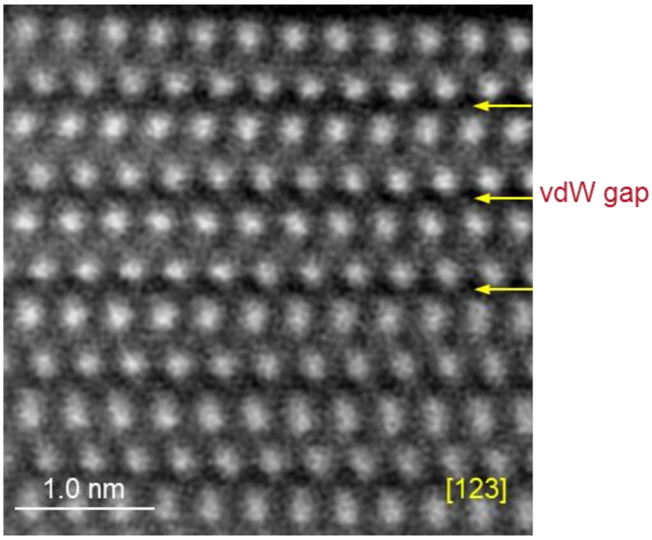

Figure S2. The recorded HAADF image in the [123] zone axis of m-GaTe.

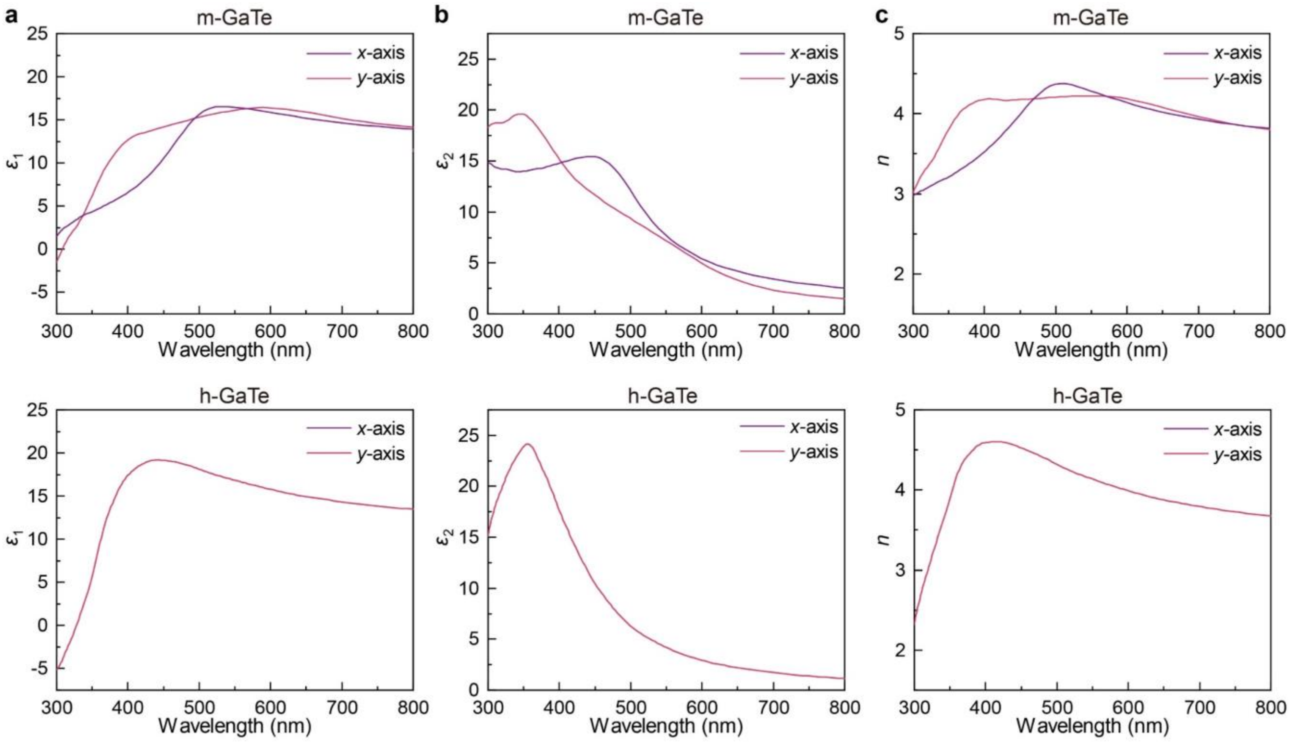

Figure S3. The DFT calculated ε_1, ε_2 and *n* profiles of bulk m-GaTe (top) and h-GaTe (bottom) along the *x*- and *y*-axis, respectively.

Table S1. DFT-computed lattice parameters of the four IV–V compounds in monoclinic (“m-”), hexagonal (“h-”), and orthorhombic (“o-”) phases. The four disordered crystals are experimentally available, but the ordered hexagonal structures are hypothetical.

	Lattice parameters				
	a [Å]	*b* [Å]	*c* [Å]	α = β [°]	γ [°]
m-GeP	15.17	9.21	3.68	90	100.37
h-GeP	3.65	3.65	15.63	90	120
m-GeAs	15.60	9.56	3.86	90	100.33
h-GeAs	3.82	3.82	15.76	90	120
o-SiP	20.51	13.79	3.52	90	90
h-SiP	3.52	3.52	15.42	90	120
m-SiAs	16.24	9.62	3.69	90	106.60
h-SiAs	3.68	3.68	15.82	90	120

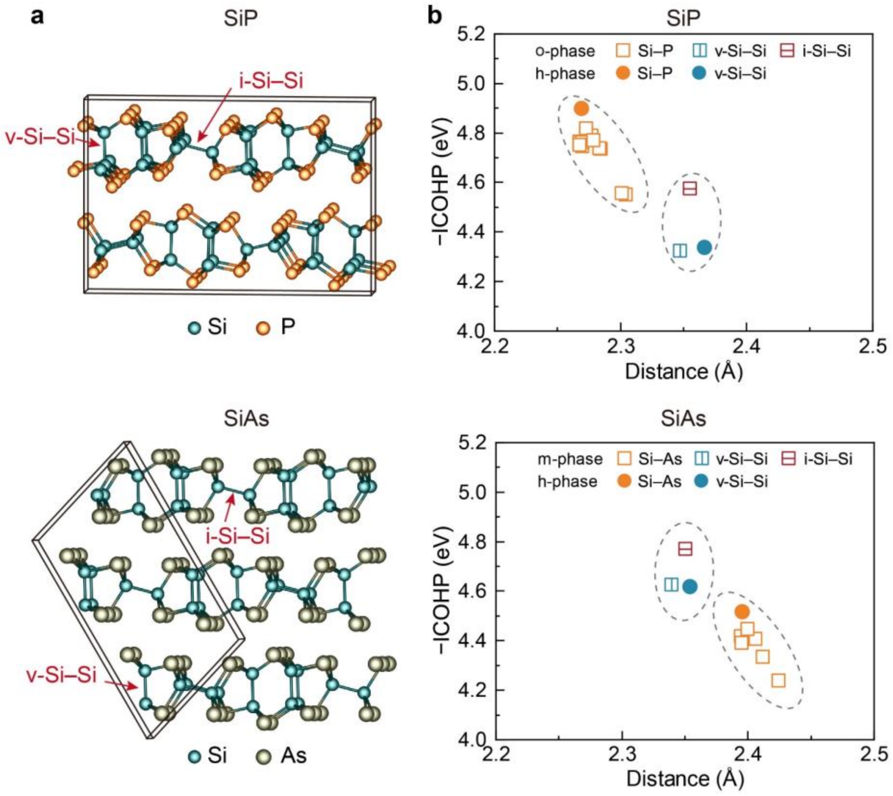

Figure S4. a) The crystal structures of o-SiP (top) and m-SiAs (bottom). b) The chemical bonding analyses of SiP and SiAs based on −ICOHP.
